# Challenges of maternity continuum of care within the primary health care in northwest Ethiopia: interpretive description using a socio-ecological model

**DOI:** 10.3389/fpubh.2024.1401988

**Published:** 2024-12-11

**Authors:** Muhabaw Shumye Mihret, Kassahun Alemu, Debrework Tesgera Beshah, Lemma Derseh Gezie, Kerstin Erlandsson, Helena Lindgren

**Affiliations:** ^1^Department of Clinical Midwifery, School of Midwifery, College of Medicine and Health Sciences, University of Gondar, Gondar, Ethiopia; ^2^Department of Epidemiology and Biostatistics, Institute of Public Health, College of Medicine and Health Sciences, University of Gondar, Gondar, Ethiopia; ^3^Department of Surgical Nursing, School of Nursing, College of Medicine and Health Sciences, University of Gondar, Gondar, Ethiopia; ^4^Department of Women’s and Children’s Health, Karolinska Institutet, Stockholm, Sweden; ^5^Department for Health and Welfare, Dalarna University and School of Health and Welfare, Dalarna University, Borlange, Sweden; ^6^Department of Health Promotion, Sophiahemmet University, Stockholm, Sweden

**Keywords:** challenges, maternity continuum of care, maternity services, interpretive description, primary health care

## Abstract

**Background:**

The maternity continuum of care plays a vital role in improving maternal and neonatal outcomes. However, its uptake remains low in Ethiopia, highlighting the need to identify challenges within the primary health care system to inform practice. Hence, this study aimed to explore the challenges of the maternity continuum of care within the primary health care system in northwest Ethiopia.

**Methods:**

An interpretive description approach was employed from March 3, 2022, to November 27, 2022, within the primary health care system in northwest Ethiopia. Maximum variation sampling was utilized, comprising 28 in-depth interviews, three focus group discussions with 29 participants, and four key informant interviews. The reflexive thematic analysis method was applied, and the results were mapped onto the constructs of the socio-ecological model.

**Results:**

The analysis identified four main themes: low maternity healthcare-seeking behavior (intrapersonal level), lack of peer and family support (interpersonal level), cultural influences on maternity care and low community responsiveness (community level), and inadequate health system readiness and response (health facility/system level). Some of the sub-themes include low health literacy and self-efficacy and misconceptions regarding maternity care at the intrapersonal level; peer and family pressure against seeking maternity care, low autonomy, and intimate partner violence at the interpersonal level; cultural influences on pregnancy disclosure and postnatal care and low social accountability at the community level; and delays in accessing ambulance services, long waiting times for maternity care, shortages of essential healthcare supplies, poor coordination of care, inadequate monitoring and evaluation, disrespectful maternity care, and dissatisfaction among healthcare workers at the health facility/system level.

**Conclusion:**

Intrapersonal, interpersonal, community, and health facility- and system-level challenges have influenced the maternity continuum of care within the primary health care in northwest Ethiopia. Since these challenges are interdependent, considering a holistic approach within primary health care could lead to an improved maternity continuum of care.

## 1 Background

Addressing maternal and neonatal mortality is a key global priority for achieving Sustainable Development Goal 3 (SDG 3) (1). While the global maternal mortality ratio (MMR) and neonatal mortality rate (NMR) are expected to drop to less than 70 per 100,000 live births and 12 per 1,000 live births, respectively, by 2030 (1), these rates remain unacceptably high in many low-and middle-income countries (LMICS), including Ethiopia. In Ethiopia, these rates are currently 401 per 100,000 live births and 33 per 1,000 live births, respectively (2), and the yearly mortality reduction rates are not at the anticipated rates to meet the global targets (2). In this regard, the maternity continuum of care (MCC), particularly when delivered through a Primary Health Care (PHC) approach, is recognized as a vital strategy to reduce these high rates of mortality (1, 3).

The continuum of care in maternity services encompasses two key dimensions: time and place. From the time perspective, the MCC involves providing care throughout the pre-intra-postnatal phases, following the recommended sequence of antenatal, delivery, and postnatal care (4). Thus, MCC is deemed incomplete if a woman misses any of the essential maternity services, such as antenatal care (ANC) contacts, facility-based delivery (FBD), or postnatal care (PNC) (4). On the other hand, the place dimension refers to the continuity of maternity care across the PHC spectrum, which includes home, community, and health facility settings (5). The place dimension assures that care is accessible and continuous across different settings, facilitating seamless transitions between home, community, and health facilities. Continuity of care across various locations ensures that women receive consistent and comprehensive support throughout their maternity journey. By integrating these two dimensions, the continuum of care in maternity services becomes more comprehensive and effective, ultimately leading to improved maternal and neonatal health outcomes. This integrated approach ensures that all aspects of maternity care are addressed, from the timing of services to the settings in which they are provided, thereby enhancing both accessibility and quality of care. Although MCC is linked to better maternal and neonatal health outcomes, its coverage remains very low in many LMICs, which carry the highest global mortality burdens. In these countries, various obstacles may hinder women’s access to MCC (6).

A Cochrane review indicates that the skills, attitudes, and behaviors of skilled birth attendants, as well as the extent to which they operate in supportive environments, significantly affect the quality of maternity care in LMICs (6). In Ethiopia, various factors can affect the uptake of MCC, including limited knowledge about maternity services and obstetric danger signs, socio-cultural beliefs, geographical and transportation challenges, shortages of medications and laboratory services in public health facilities, and instances of rude or abusive care (7–8). Given the multi-dimensional and complex nature of these challenges, applying a socio-ecological model (SEM) could provide a comprehensive understanding of the intricate health issues and patterns at different levels (9). The SEM is a holistic framework that emphasizes the interconnected relationships within healthcare systems, moving beyond single-level influences to consider the interactions of multiple elements in the social environment, thereby offering deeper insights into the studied issues (10, 11).

While some studies have explored the challenges of MCC from a time perspective (7–8), there is scarce evidence regarding the challenges from the place dimension within PHC in Ethiopia. Furthermore, previous research has often focused on challenges related to antenatal care, facility delivery, or postnatal care separately (7, 8, 12–15). However, it is crucial to consider MCC as a whole and explore the various challenges affecting it, including the place dimension, to inform policy and practice. Additionally, there is a significant demand for PHC-oriented research, one of the ten operational levers of PHC (16). Therefore, this research aims to comprehensively explore the multi-dimensional challenges of MCC, treating it as an integrated entity and considering the place dimension within the context of frontline PHC in northwest Ethiopia.

## 2 Methods and materials

### 2.1 Qualitative approach and research paradigm

We embraced the constructivism philosophical paradigm, which underscores the critical role of context in the construction of knowledge (17). This paradigm also directs us in choosing appropriate alternative approaches and methods that are consistent with its principles. As such, we utilized the Interpretive Description (ID) qualitative approach (18) within the framework of qualitative research methodology. Drawing inspiration from grounded theory, naturalistic inquiry, ethnography, and phenomenology (19), ID directs data analysis toward exploring wider ranges of healthcare perspectives (20). We chose ID as it provides a coherent strategy for conducting research in clinical settings, health sciences, and educational contexts and is highly adaptable, making it well-suited for exploring complex phenomena in healthcare practices (18). Its practical applicability and depth of insight have led to its growing popularity among researchers in various health disciplines (19, 21, 22).

### 2.2 Study settings

The study was conducted within the PHC in Amhara National Regional State, Ethiopia. Ethiopia’s healthcare system is organized into three tiers: primary, secondary, and tertiary care levels. The PHC unit consists of a network of primary hospitals, health centers, and health posts, which form the foundation of primary-level healthcare structure (2). The frontline PHC specifically includes health centers and their affiliated health posts (2, 23). Typically, each Woreda (district) has an average of five frontline PHC units, with each unit comprising around five satellite health posts—the smallest village-level health facility—along with a referral health center (2).

To strengthen human resources in PHC, Ethiopia launched the Health Extension Program (HEP) in 2004 (24). This initiative involved establishing a health post in each Kebele (the smallest administrative division) and assigning two female health extension workers (HEWs) to provide PHC services at the health posts and in a home-to-home approach. The Women’s Development Army (WDA), established in 2010, serves as a key community-based structure supporting HEWs in Ethiopia (24, 25). The WDA operates through a one-to-five network and further bottom-up women’s connections, where a small group of women are organized to facilitate health-related initiatives, particularly in reproductive, maternal, newborn, and child health (25). A notable part of frontline PHC initiative is the monthly Pregnant Women’s Conference (PWC) (26), which functions as an outreach and community dialogue platform. Held at the local Kebele level, the PWC involves various stakeholders, including a midwife, HEW(s), and maternity service end-users like pregnant and postnatal women. Sometimes, other participants, such as WDA members, supervisors of HEWs, and health center heads, join the conversation to address critical health issues. This collaborative effort aims to improve health outcomes by integrating community support into PHC services (2, 27, 28).

Within the PHC, maternity services, including ANC, labor and delivery care, and PNC, are primarily delivered by midwives. However, when the number of midwives is limited, other healthcare workers, such as nurses and health officers, step in. Additionally, HEWs are expected to provide PNC at health posts and/or in the home. Assisted by leaders of the WDA in the village and guided by midwives, HEWs carry out various health promotion activities and community mobilization efforts for maternal and child health (MCH) programs (24). This network among midwives, HEWs, and leaders of WDA is also supervised by designated personnel from the health center and district health office. In addition, there are several maternity care delivery platforms or programs within the PHC, including maternity waiting homes (MWHs), the 24-h stay at facility after delivery, free maternity ambulance service, and the minimum of eight ANC contacts (ANC8+) model (26, 29).

### 2.3 Study participants and sampling technique

This study included participants from four districts—Gondar Zuria, West Dembiya, East Dembiya, and Wogera—in the Central Gondar zone from March 3, 2022, to November 27, 2022. When choosing the districts, we took into account the issues of representativeness, security, and feasibility. In addition, representatives from the Amhara national regional level and the central Gondar zonal level took part in the study. We used the maximum variation sampling technique to capture the multi-dimensional and widest range of perspectives on the challenges of the MCC within the PHC. As such, we included end-users (i.e., pregnant and postnatal women); their significant others (i.e., mothers, mothers-in-law, and husbands); community representatives (i.e., traditional birth attendants (TBAs), leaders of WDAs, and other opinion leaders); maternity service providers (i.e., midwives and HEWs); and MCH program managers (i.e., Woreda health office managers and zonal and regional MCH coordinators). In total, 61 participants were included in the study: 28 through in-depth individual interviews (IDIs), 29 through focus group discussions (FGDs), and 4 through key informant interviews (KIIs).

### 2.4 Data collection

We used IDIs, KIIs, and FGDs for data collection. Accordingly, we undertook 28 IDIs (8 with midwives, 4 with HEWs, 7 with pregnant mothers) (two of them were also leaders of WDA), 3 with postnatal women, and 6 with significant others (i.e., four of them were also TBAs and the remaining two were husbands); 4 key informant interviews (2 with district health office managers, 1 with the zonal MCH coordinator, and the remaining 1 with the regional MCH coordinator); and 3 FGDs involving 29 participants, including leaders of WDA, TBAs, and community opinion leaders. Individual (Supplementary File S1) and FGD (Supplementary File S1) interview guidelines were prepared with open-ended questions and probes. All IDIs and FGDs had been undertaken in a face-to-face manner, either at the health centers or health posts, taking the participants’ convenient place of choice into consideration. In addition, two KIIs (i.e., those taken place with woreda health office managers) were undertaken in a face-to-face way at their own offices, while the remaining two KIIs (i.e., those undertaken with zonal and regional MCH coordinators) were conducted through telephoning by taking their convenient time (i.e., they chose evening time as they reasoned out that they were so busy at daytime) into account. No study participant refused to participate. The data collection was undertaken primarily by the MSM along with two research assistants. No one else was present besides the participants and researchers. All interviews were tape-recorded with study participants’ informed consent. The sessions took 13–71 min each, and no repeat interview was made as each of the interviews was clear. In addition, important field notes were made during and/or after the interviews.

### 2.5 Data analysis

**Figure 1:**
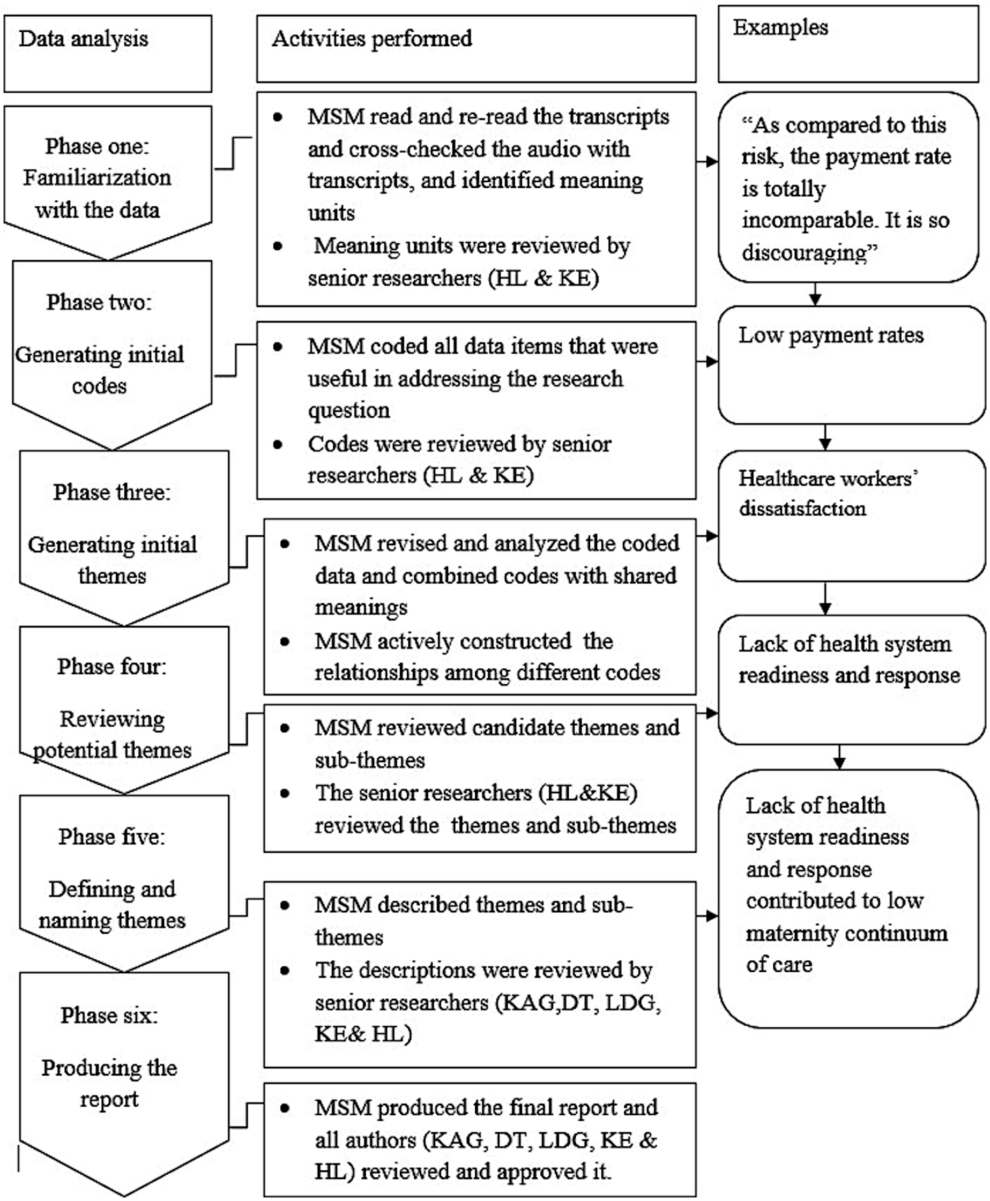
Schematic representation of reflective thematic analysis for challenges of maternity continuum of care within the primary healthcare of northwest Ethiopia (21).

We employed a reflexive thematic analysis method (30–31). All audio recordings were transcribed verbatim, translated into English, and imported into ATLAS.ti version 7.1.4 for analysis, with field notes also included. The overall analytic process followed six key phases: (1) familiarization with the data, (2) generating initial codes, (3) identifying initial themes, (4) reviewing potential themes, (5) defining and naming themes, and (6) producing the final report (30, 31) (Figure 1). During the initial phase, the first author reviewed the transcripts multiple times while concurrently listening to each audio recording and comparing it to its corresponding transcript. In the second phase, the first author identified sections of the transcripts that are relevant for the development of sub-themes and themes. As such, any piece of data containing useful information related to the research questions was coded. As a result, two sets of codes emerged, and a hybrid coding approach (i.e., both deductive and inductive) was employed. This included codes similar to those identified in previous studies (7–8) (deductive coding) and new codes that uniquely emerged in this study (inductive coding). In the third phase, the first author, along with input from the fifth and last authors, grouped similar codes and organized them into related categories, refining them into sub-themes and overarching themes through several rounds of revisions (30, 31). In the fourth phase, the first author reviewed the potential themes and sub-themes generated during the third phase (31). This process involved revisions based on feedback from the fifth and last authors. Additionally, the first author shared the results with the entire research team (31). Authors reached consensus on the final themes and sub-themes after incorporating the team’s feedback and making iterative adjustments (30, 31). In the fifth phase, the first author interpreted, described, and defined each team and sub-theme based on the dataset and research questions and shared them with all research team members. In consultation with other authors, the first author then mapped the results onto the constructs of the SEM (9, 11) as transferring the generated themes and sub-themes onto the SEM would aid in a comprehensive understanding of the study results (31). As such, we utilized the SEM as adapted by McLeroy et al. (9) which identifies five levels of influence specific to health behaviors: intrapersonal factors, interpersonal factors, community factors, institutional factors, and policy factors (11). This model is highly adaptable and can be tailored to suit the distinct phenomena of various studies (9, 11).

**Figure 2:**
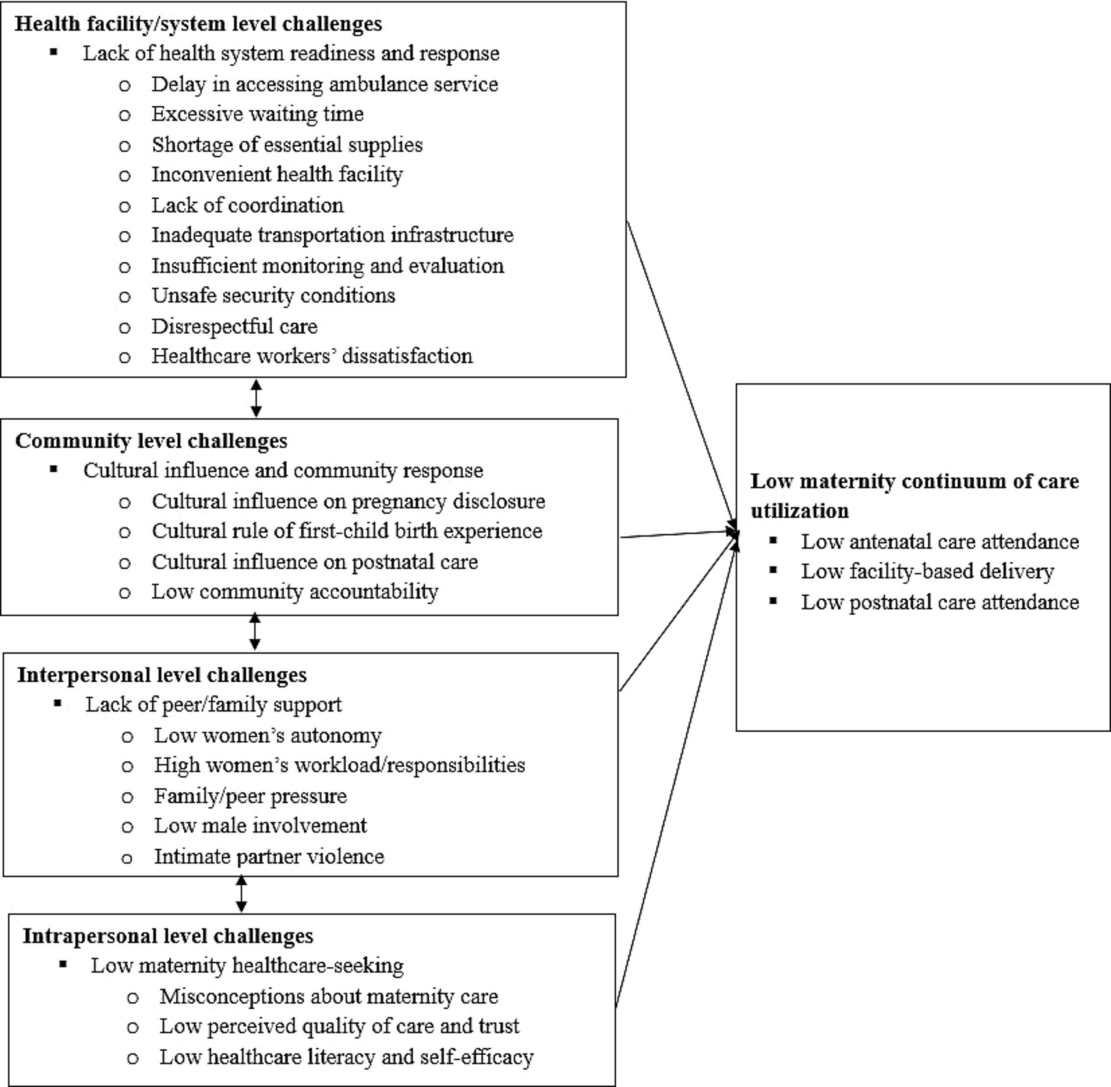
Theoretical framework based on socio-ecological model for challenges of maternity continuum of care within the primary health care of northwest Ethiopia.

In our study, we further adapted the SEM into four levels by merging institutional and policy factors, as our findings did not provide a clear distinction between these two levels. Therefore, the four-level SEM constructs in our study include intrapersonal, interpersonal, community, and health facility/system/policy level challenges (Figure 2). At the base level, intrapersonal factors encompass maternity service end-users’ knowledge, attitudes, confidence, and perceptions toward maternity services (11). The second level, interpersonal challenges, includes intimate-relational factors such as the influence of significant others (e.g., male partners, parents, parents-in-law, and peers) on the uptake of maternity services by end-users (32). The third level focuses on community influencers, which involve cultural norms, beliefs, and practices shared within the community, as well as responses to these challenges from community opinion leaders (11). The fourth level comprises health facility/system factors, including healthcare provider-related factors, infrastructural and physical factors, security issues, modes of maternity service delivery and guidelines, availability and accessibility of essential services, and national policy and commitment toward maternity healthcare (11). Thus, all generated themes and subthemes were mapped on either of these four SEM domains. In the last stage, the first author compiled a final report and distributed it to the research team members for approval (31). The results and all other sections were reported guided by using the consolidated criteria for reporting qualitative research (COREQ) (33) (Supplementary Table S1).

### 2.6 Reflexivity

The first author, a male researcher and an assistant professor of clinical midwifery, holding a bachelor’s degree in midwifery and a master’s degree in clinical midwifery, conducted the data collection and analysis. The second author, a male PhD-holding researcher and professor of epidemiology and public health; the third author, a female PhD-holding researcher and an assistant professor of surgical nursing; and the fourth author, a male PhD-holding researcher and associate professor of biostatistics, reviewed sub-themes and the final report. Moreover, the fifth and sixth authors, female PhD-holding researchers and professors who hold midwifery and nursing licenses, undertook an iterative review of the analysis and reports. All authors have previous experience in conducting qualitative research studies, involving individual interviews and focus group discussions.

## 3 Results

### 3.1 Participants’ characteristics

About 44 (72.1%) and 48 (78.7%) of the participants are females and rural dwellers, respectively. At the time of the interview, 19 (31.1%) and 4 (6.6%) of the participants were pregnant and postnatal, respectively. The majority (60.7%) of the participants were not able to read or write. A large proportion (70.5%) of the participants were farmers. Nearly half (47.5%) of the informants were under the category of community representatives (Table 1).

**Table 1:** Characteristics of study participants for challenges of maternity continuum of care within the primary health care of northwest Ethiopia (*n* = 61).

Variable	Number	Percent
Age		
<35	29	47.5
≥35	32	52.5
Educational status		
Unable to read or write	37	60.7
Formal education (grade 1–12)	7	11.5
Diploma or above	17	27.9
Marital status		
Currently in marriage	57	93.4
Currently not in marriage	4	6.6
Occupation		
Farmer	43	70.5
Government employee	16	26.2
Merchant	2	3.3
Status in the reproductive cycle		
Pregnant	19	31.1
Postnatal	4	6.6
Non-perinatal	21	34.4
Not appropriate (male)	17	27.9
Major representative category		
End- users	10	16.4
Significant others	6	9.8
Community representatives	29	47.5
Maternity service providers	12	19.7
Program managers	4	6.6
Special role within the primary health care		
Leaders of women’s development army	7	11.5
Traditional birth attendant	7	11.5
Midwifery practitioner	8	13.1
Health extension worker	4	6.6
Woreda health office manager	2	3.3
Zonal maternal and child health manager	1	1.6
Regional maternal and child health manager	1	1.6
Other community’s opinion leader	22	36.1
End-user or significant other (with no additional special role)	9	14.8

### 3.2 Key findings

Four main themes emerged from the study. These include: (1) low maternity healthcare-seeking behavior (intrapersonal level); (2) lack of peer/family support (interpersonal level); (3) cultural influence on maternity care and low community responsiveness (community level); and (4) lack of health system readiness and response (health facility/system level). The four global themes with subthemes are presented below with quotes (Table 2).

**Table 2:** Sub-themes and themes nested to socio-ecological model constructs.

Sub-themes	Themes	Socio-ecological model
Misperceptions about maternity services	Low maternity healthcare-seeking behaviors	Intrapersonal level
Low perceived care quality and mistrust in providers		
Low health-literacy and self-efficacy		
Low women’s autonomy	Lack of peer/family support	Interpersonal level
High women’s workload and responsibilities		
Family/peer pressure against maternity care		
Low male’s involvement		
Intimate partner violence		
Cultural influence on pregnancy disclosure	Cultural influence on maternity services and low community’s responsiveness	Community level
Cultural rule of first-childbirth experience		
Cultural influence on postnatal care		
Low social accountability		
Delays in accessing ambulance service	In adequate health system readiness and response	Health facility/system level
Excessive waiting times for maternity care		
Shortage of essential healthcare supplies		
Inconvenient health facility environment		
Lack of coordination for maternity care		
Inadequate transportation infrastructure		
Insufficient monitoring and evaluation		
Unsafe security conditions		
Disrespectful maternity care		
Health workers’ dissatisfaction		

#### 3.2.1 Low maternal healthcare-seeking behavior (intrapersonal level)

Low maternal healthcare-seeking behavior, as a result of misperceptions about maternity services, low perceived care quality and mistrust in providers, and low health literacy and self-efficacy, emerged as one of the prominent challenges of MCC.

##### 3.2.1.1 Misperception about maternity service

Service end-users often hold the misconception that quality healthcare is synonymous with receiving medications, leading to a preference for medicalized over de-medicalized maternity care. Their satisfaction with health facilities is frequently linked to being given medicines, injections, or intravenous fluids. This belief, reinforced through community conversations, has created a cultural expectation where women are asked about the treatments they received at health facilities. In some cases, women even express a desire for medications to take home in order to meet these expectations. When no medication is provided, dissatisfaction can arise, which negatively impacts their perception of maternity care quality and reduces their adherence to the MCC. However, the consistent provision of iron-folic acid supplements has been found to positively address these medicalization-related expectations within the community. *“Clients aren’t satisfied unless you prescribe any medication. The good thing now is that we give them this Iron 90+ as soon as they come, and they see it as a gift.” Key informant* interview participant, program manager.

Similarly, women in labor at health facilities often expect to receive intravenous fluids, regardless of their medical condition. When this expectation is not met, they may express dissatisfaction with the care provided during labor and delivery. This dissatisfaction can negatively impact their overall perception of maternity services, ultimately reducing their willingness to utilize such services in the future. “*In our community, women in labor anticipate receiving intravenous fluids disregarding their health condition. Hence, they start complaining with the service unless they receive those fluids.”* In-depth individual interview participant, service provider.

##### 3.2.1.2 Low perceived care quality and mistrust in providers

Some women may have a low perceived quality of care, often resulting from previous negative childbirth experiences or a lack of trust in healthcare providers’ competency. This perception increases the likelihood of incomplete utilization of MCC. While regular use of maternity care is generally expected to result in positive childbirth experiences, there are occasional instances where even women who consistently adhere to these services face adverse birth outcomes. Such negative experiences can weaken community trust in the quality of maternity care, leading to reduced motivation for future service use. This decline in trust may foster rumors and negative perceptions, further lowering health-seeking behavior and negatively impacting maternity service programs. *“Mothers who experienced poor maternal or neonatal outcomes while they got delivered at health facilities felt disappointment and lost their motivation to comply with maternity services for subsequent pregnancies.”* In-depth individual interview participant, service provider.

Participants noted that some healthcare providers lacked the competence to accurately diagnose pregnancy, leading to a loss of trust in their services. They recommended that these providers enhance their skills to properly assess and diagnose pregnancy and labor, ensuring appropriate clinical decisions. *“Let me share my notice with you. We brought my daughter to the health facility as she complained of labor pain. Upon arrival, a healthcare provider evaluated her, and we waited for more than 8 h there. Finally, he told us as if true labor was not initiated and informed us to go back to home. However, she experienced severe labor pain and was delivered at home upon arrival of my brother’s home in the town, and we brought her back to the health facility for evaluation. So, I was disappointed that he did not identify her condition correctly.”* Focus group discussion participant, community representative.

##### 3.2.1.3 Low health literacy and self-efficacy

In rural areas, some women have low health literacy and self-efficacy, resulting in a limited understanding of the importance of timely initiation and follow-up for maternity services. They often lack the confidence to follow healthcare recommendations and tend to seek care only when complications arise or to confirm pregnancy. This combination of low health literacy, self-efficacy, and adherence, along with the influence of traditional beliefs, presents a significant challenge and contributes to the incomplete utilization of maternity services. *“Only a few women have favorable awareness; they come to health facilities only when they get sick or just to confirm whether the symptoms they feel are related to pregnancy or not. Similarly, in the intrapartum period, the women seek healthcare and come to health facilities only when they experience complications. So, I believe that the main problem is related to the awareness gap; there is no doubt.” Key informant interview participant, program manager.*

Participants noted that women’s low health literacy and self-efficacy are largely due to inadequate community awareness efforts. They explained that community health education programs often fail to reach the majority of rural communities. Even where health education programs are implemented, they tend to address only nearby communities, and these efforts are insufficient to achieve the desired behavioral changes. *“Our community remains uneducated, and we do not reach out to them to educate. We go for reducing maternal and neonatal deaths. But our efforts are limited to the nearby communities within a 15–20 km radius. Even in these nearby communities, we observe big awareness problems.”* Key informant interview participant, program manager.

#### 3.2.2 Lack of peer/family support (interpersonal level)

In this study, the lack of peer and family support encompasses several factors: low women’s autonomy, high workloads and responsibilities for women, family and peer pressure against seeking maternity care, low involvement of males, and instances of intimate partner violence.

##### 3.2.2.1 Low women’s autonomy

Participants observed that in certain communities, many women have limited empowerment and decision-making authority concerning their healthcare. These women often lack autonomy and empowerment in pregnancy-related matters, leading to situations where their male partners or mothers-in-law make health decisions for them. This restricted autonomy and empowerment result in incomplete utilization of maternity services, as their capacity to make independent health decisions is hindered by external influences. *“But still, there are very ordinary males with very poor attitudes. They want to oppress and restrict wives from any social interaction or healthcare services. As a result, women remain with low empowerment and decision-making power.”* In-depth individual interview participant, service provider.

##### 3.2.2.2 High women’s workloads and responsibilities

End-users reported that their heavy home and farming workloads often prevent them from attending maternity services. In rural communities, women typically juggle demanding roles such as food preparation, farming, and caring for domestic animals, along with the responsibility of looking after children who are in school. These extensive responsibilities create significant burdens, leading perinatal women to delay scheduling antenatal care (ANC) appointments and struggle with interim follow-ups. These challenges contribute to the incomplete utilization of maternity services. End-users reported that their heavy home and farming workloads often prevent them from attending maternity services. In rural communities, women typically juggle demanding roles such as food preparation, farming, and caring for domestic animals, along with the responsibility of looking after children who are in school. These extensive responsibilities create significant burdens, leading perinatal women to delay scheduling ANC appointments and struggle with interim follow-ups. These challenges contribute to the incomplete utilization of maternity services. *“I came here for my first ANC contact at 7 months of gestation due to my high workload at home… We are so busy with home and farming workloads. We also look after domestic animals as we send our children to school. We have no free time for maternity service attendance.”* In-depth individual interview participant, end-user.

##### 3.2.2.3 Family/peer pressure against maternity care

Significant others, such as key individuals who influence women’s decisions about maternity healthcare, play a crucial role in the utilization of these services. Study participants highlighted that in some communities, mothers-in-law often discourage the use of maternity services. Narratives from older female family members, who typically describe pregnancies without complications, foster skepticism about the need for maternity care. This skepticism, in turn, leads to delays in seeking, accessing, or receiving maternity care among perinatal women. *“Especially the influences and impacts of mothers-in-law in the maternity services are too much. Mothers-in-law narrate their traditional beliefs and practices that will negatively affect women’s decision to attend as per the standard ANC care… Mothers-in-law discourage pregnant women and narrate that they had not visited health facilities while they gave about twelve and thirteen children*.*” Key informant interview participant, program manager.*

In some communities, peers, including neighbors and friends, may also discourage the use of maternity services. Additionally, some perinatal women may place more trust in the advice from their social networks and family than in the guidance of healthcare providers. *“There are also others who discourage mothers against coming to health centers; these include peers such as neighbors and friends. These people advise her as if ANC follow-up and other maternity services had no importance for her. Consequently, even a woman who has already planned to come to the health center will then cancel out her plan.”* In-depth individual interview participant, service provider.

In many rural communities, male partners often continue to discourage their wives from visiting health facilities for maternity services. This negative influence is particularly pronounced during the summer season and harvest times when men are fully occupied with outdoor farming activities. “E*specially in the summer season, males spend most of their time doing outdoor activities. During that time, when we identify an eligible mother for maternity waiting home service and counsel her to use the service, she does not agree. Even the husband insists his wife return home soon. For a husband, his home is dark without his wife, and a wife is aware of that. As a result, women do not agree to use maternity waiting home.”* In-depth individual interview participant, service provider.

##### 3.2.2.4 Low males’ involvement in maternity services

Participants believe that increased male involvement in maternity care—such as providing practical support in healthcare planning, visiting health facilities, and accompanying women—could make a significant difference, as men are often the primary decision-makers and heads of households in many communities. Despite the potential benefits of involving male partners in maternity care, participants noted that this involvement remains low and challenging to achieve. This lack of male participation continues to contribute to low coverage of the MCC. “*Involving husbands in ANC is challenging, from my experience.”* In-depth individual interview participant, service provider.

##### 3.2.2.5 Intimate partner violence

Intimate partner violence during pregnancy persists, involving physical, sexual, or psychological harm inflicted by husbands or male partners. Some perpetrators prevent victims from engaging in social events or seeking maternity services, fearing disclosure of violent episodes. Perpetrators closely monitor and restrict victims’ interactions, using physical force if victims attempt to meet others or access healthcare services. This violence results in victims staying at home, avoiding maternity services, and enduring such circumstances throughout their lives. *“Let me share my experience with you. I went to one of the Kebeles in our catchment area for community health insurance purposes with security staff. At that time, I got the opportunity to talk with one pregnant mother. She had four children and is currently pregnant. She told me that she had no ANC contact for the current pregnancy. She also told me that she delivered all children except the youngest child at home. She mentioned that her husband does not allow her to attend for maternity services. During our conversation, her husband came to us and started committing verbal abuses. He continued insulting us. If I was not with security staff, he even would use physical force to damage me.”* In-depth individual interview participant, service provider.

#### 3.2.3 Cultural influence on maternity care and low community’s responsiveness (community level)

This section explores the cultural influences on maternity services, including norms around pregnancy disclosure, traditional expectations for the first childbirth experience, and cultural practices related to postnatal care. It also addresses the low level of community accountability in confronting and addressing these negative cultural influences.

##### 3.2.3.1 Cultural influence on pregnancy disclosure

Pregnant women avoid disclosing their pregnancy status early due to cultural beliefs, considering it a cultural taboo. Traditional views dictate that early disclosure is discouraged as there is uncertainty about the pregnancy’s continuation. Fear of early disclosure to the community leads to delayed initiation of ANC. This delay, driven by traditional and cultural beliefs, contributes to the incomplete utilization of maternity services on the continuum. *“Most of them initiated ANC lately, and they reasoned it out that the offspring shall mature in a hidden fashion before the community becomes aware of it as per the cultural and traditional beliefs… I think starting ANC timely for them is just like a traditional taboo.”* In-depth interview participant, service provider.

Women indicated that they follow cultural norms of keeping their pregnancy status private and often feel embarrassed to disclose it. As a result, there are times when pregnant women leave health facilities without receiving maternity services due to their reluctance to reveal their pregnancy status. This shyness leads to delayed disclosure of their pregnancy and later booking of ANC appointments. *“I’m so shy; I even return back without getting the services after coming to this health facility because I felt shy. I cannot express my feelings and ideas in front of others.”* In-depth individual interview participant, end-user.

##### 3.2.3.2 Cultural rule of first-childbirth experience

Traditional beliefs dictate that women should adhere to their first pregnancy and childbirth experiences. According to these beliefs, if a woman had not sought maternity services in her initial pregnancy, she should continue the same practice in subsequent pregnancies. Choices such as the type of transport used and the place of delivery are influenced by these views. People in these communities believe that deviating from these practices may lead to health problems in the current pregnancy. Consequently, some women conform to these cultural beliefs, resulting in incomplete utilization of maternity services. *“Traditionally, pregnant women tend to adhere to their experiences from their previous childbirths. For example, if a woman had never traveled by bus during her first childbirth process, she would not use a bus for subsequent childbirths*. In-depth individual interview participant, service provider.

##### 3.2.3.3 Cultural influence on postnatal care

Traditional practices in the community are especially noticeable during the postpartum period. In many communities, postpartum women are expected to remain in bed for at least the first 10 days after childbirth. A prevalent cultural belief, the “10th day of postpartum” rule, dictates that a postpartum woman must remain in bed until the 10th day, not even staying home alone. A family member, preferably a male, is designated to care for her, or a metallic object is placed by her side if no one is available. The belief suggests that leaving the woman alone before the 10th day may lead to evil spirit-facilitated mental disorders.

*“From the outset, no postnatal woman comes down from the bed before the 10th day of postpartum… she has never been left alone. Otherwise, she will experience crazyness with severe mental disease that can result in madding. So, attending for postnatal care before the 10th day is culturally unacceptable and infeasible in our community.”* In-depth individual interview participant, end-user.

##### 3.2.3.4 Low social accountability for maternity services

In many communities, influential figures like community opinion leaders, religious leaders, and others play a significant role in maternity services, often with widespread acceptance. Their impact can lead the community in positive or negative directions, serving as change agents. While some actively contribute to positive change, others are less responsive, adhering to traditional beliefs rather than challenging harmful practices. In communities with unresponsive representatives, home deliveries and associated risks, such as maternal and neonatal deaths, are prevalent. *“My mother died after she had been experiencing vaginal bleeding for two weeks, and no one could help and brought her to a health facility. The community members and traditional birth attendants strictly adhered to traditional beliefs and practices.”* Focus group discussion participant, community representative.

#### 3.2.4 Lack of health system readiness and response (health facility/system level)

Lack of health system readiness and response represents significant challenges at the health facility or system level that deter women from utilizing maternity care services. These challenges include: delay in accessing ambulance service, excessive waiting times for maternity care, shortage of essential healthcare supplies, inconvenient health facility environment, inadequate transportation infrastructure, poor coordination for maternity care, insufficient monitoring and evaluation, unsafe security conditions, disrespectful maternity care, and healthcare workers’ dissatisfaction.

##### 3.2.4.1 Delay in accessing ambulance service

Study participants narrated that inaccessibility to the ambulance services has contributed to phase two and three obstetric delays. The issue is deep-rooted, extending from ambulance drivers to health office managers, and has led to maternal and neonatal deaths. Stakeholders, including drivers and managers, often exhibit misconduct, irresponsiveness, and arrogant behavior, with reported abuse of ambulance cars for personal interests over maternity service. *“After tremendous efforts, the ambulance came here and brought her [the laboring woman] to Gondar too lately. Finally, she passed away due to those unreasonable delays. This is the iceberg of many problems that occurred due to deep-rooted problems related to ambulance services. No one hears you if you underpin the seriousness of such an issue*.” In-depth individual interview participant, service provider.

The misuse of ambulance services is a prevalent issue, with vehicles often being used for non-maternity purposes. Participants indicated that inadequate ambulance services might stem from low incentives and a shortage of drivers. They recommended increasing drivers’ salaries and the number of drivers at each health center to address these problems. *“From the beginning, the ambulance has to be ready for 24 h’ maternity service; the next issue is that using the ambulance for other purposes is abusing and a crime… The ambulance drivers’ incentive issue is also concerning, just like the healthcare professionals’ incentive and payment issues. We know that unless the ambulance drivers go to the rural village and bring the pregnant and/or laboring women to the health facilities, healthcare providers will not provide the quality maternity services. Hence, the role of drivers in relation to quality maternity service is high, and we need to consider their incentives and improve their satisfaction as well. So, the number of ambulance drivers per health facility should be at least two in number, as one driver could not be active for 24 h*.” Key informant interview, program manager.

The impact of the internal conflicts in Ethiopia on maternity services varies, with instances where ambulance drivers become victims of ethnic conflicts, leading to frustration among drivers and limiting their ability to freely serve the community. Internal conflicts lead to the destruction of significant resources, including ambulance cars, health facilities, and infrastructure. Consequently, the coverage and quality of maternity services in conflict-affected communities are severely impacted. *“We know we lost huge resources, including more than a hundred ambulances, during the internal conflict.”* Key informant interview participant, program manager.

##### 3.2.4.2 Excessive waiting times for maternity care

At most health centers, lengthy waits at the chart room for chart services for meeting maternity providers, undergoing basic tests, and obtaining essential drugs impede timely access to care. The traditional approach of storing and searching charts in the chart room is a prominent but often overlooked barrier to maternity service utilization. Maternity service providers uncovered that undue long waiting time from chart room is one of the issues that become the barrier for quality maternity care. *“In our health facility, the big challenge was occurred especially at the chart room. There was a time consuming and a bureaucratic process in the chart room that everybody was complaining with that traditional platform”* In-depth individual interview participant, service provider.

Service users also complained that they suffered from unfair bureaucratic processes for receiving chart and other services in connection to maternity services. They noted that many women leave without receiving services, driven away by prolonged waiting times and procedures, contributing to a decline in the MCC. *“I’m so sorry for them! I suffered from many bureaucratic processes here. Many women are suffering from such similar processes. For example, I come here last week on Friday day then they appointed me for Tuesday, I spent my full time on Tuesday for nothing; I have not received any service on Tuesday too! And Today I come at the very early morning. But still (Mid-day) I have not received the service. If they give me the service today, it will be alright. If not, I’ll never come again. They forced us to go back and forth for nothing here and there. They neither give services here timely nor refer to other health facilities. They simply insulted us here. I wish I did not come here.”* In-depth individual interview participant, end-user.

Healthcare providers reported that, despite using a first-come, first-served approach for delivering maternity services, unacceptable delays in the chart room drive women away from continuing with maternity care. They have observed that many pregnant women return home without receiving the necessary services, primarily due to issues in the chart room. *“In this health center all are treated in the first come first serve principle, there is no especial groups who get priority… There are pregnant mothers returning back without receiving services mainly due to this problem (at chart room). I have observed that about five mothers went to home without getting maternity services in a single day at this health center. There are even larger similar hidden scenarios”* In-depth individual interview participant, service provider.

Maternity service providers also identified long waiting times in the laboratory room as a major barrier to effective maternity care. They recommended setting up separate laboratory rooms for mothers and infants during the perinatal period to address this issue. *“Mothers spend their time mainly at laboratory, you know hematocrit, blood group, hepatitis, stool, urine* etc. *will be requested to be done. As there is no a separate laboratory for mothers and as crowded cases come from OPD for laboratory services, they wait long time at the laboratory. There is a big problem…I think, if a separate laboratory service is arranged for mothers or if one laboratory professional assigned for only mothers, this problem will be resolved.”* In-depth individual interview participant, service provider.

Excessive waiting times for charts or other services can damage the relationship between service providers and users. Negative interactions arising from these delays create a poor impression, which can significantly affect future follow-ups. *“The challenge is, for example, I come here at morning for antenatal check-up; no one follows me and still I have not received a service. So, we agreed that we shall not go to the health facilities because the healthcare providers do not treat us properly”* In-depth individual interview participant, end-user.

##### 3.2.4.3 Shortage of essential healthcare supplies

In many health centers, the lack of essential drugs, equipment, and supplies is a common problem that can undermine the quality of maternity services. Although Ethiopia has a policy for free maternity care, public health facilities often lack these crucial resources. Consequently, many perinatal women are compelled to buy them from private providers or face unnecessary referrals. This inaccessibility negatively impacts the quality of maternity care. *“Most of the time, we are ordered to purchase drugs from the private pharmacy or to get laboratory services out of this health center.”* In-depth individual interview participant, service user.

Delays in responding to requests for replenishing stock-out essential drugs, along with the inaccessibility or unavailability of these crucial medicines, contribute to incomplete provision of maternity services and lead to unnecessary referrals to higher health facilities in many centers. *“There is shortage of inputs for maternity services, and thus women may not receive complete care on the same day and will be appointed for a week. So, stakeholders such as the woreda health office should closely follow and fill gaps timely. For example, in our health center, we midwives report the inputs to be fulfilled timely. However, for a for a long time, more than two weeks could take to refill. As a result, we refer the clients to other health facilities.”* In-depth individual interview participant, service provider.

##### 3.2.4.4 Inconvenient health facility environment

The delivery room and maternity waiting home are intended to offer a home-like environment to encourage clients to use health facilities and reduce home deliveries. Social ceremonies, such as coffee and porridge ceremonies, which are typically held at home during home deliveries, are expected to be conducted at health facilities with community-contributed inputs. However, participants reported that there are still gaps in creating this home-like atmosphere at health facilities, and improvements are needed. *“In addition, we observed problems while we brought laboring mothers to the health facility. We expect social services like coffee and bread ceremonies as we collect money from the community for that purpose. However, they do not give us such social services regularly. This could bring about dissatisfaction and demotivation for the community.”* Focus group discussion participant, community representative.

Similarly, creating a homelike environment at health posts during women’s conferences is expected to encourage and sustain women’s participation in the maternity continuum of care. However, current efforts in this area have declined, leading to complaints and diminishing clients’ active engagement in their healthcare. *“They have also had the experience of enjoying a ceremony, including a coffee ceremony at the health post, on the day of the women’s conference. But these positive experiences got declined this time, and women are complaining of this.”* In-depth individual interview participant, service provider.

The lack of sustainable access to basic amenities like water and electricity at health facilities caused inconvenience for clients. A common complaint is the non-sustainable and interrupted electricity sources, leading to unnecessary delays for services such as laboratory tests. This inconvenience contributes to low client satisfaction. Participants recommended implementing backup mechanisms, such as generators, to ensure uninterrupted provision of maternity services. *“One thing that needs to be considered is the issue of light. We wait too long for laboratory services due to the “on-and-off” pattern of light. They could set up a backup light source; they could have a generator as a backup for the light source.”* Focus group discussion participant, community representative.

##### 3.2.4.5 Lack of coordination for maternity services

One of the commonly reported challenges is the lack of coordination within the home-community-health facility ecosystem. In Ethiopia’s current PHC system, this includes various actors and platforms such as leaders of WDA (community volunteers at the village level), HEWs (community-level healthcare workers), and midwives and other skilled health personnel at health centers and primary hospitals. Additionally, supportive personnel like ambulance drivers, program managers, and other stakeholders play crucial roles in maternity care. Participants highlighted that inadequate coordination among these various actors remains a significant issue. This lack of coordination can lead to duplicated efforts, delays in accessing essential services, low uptake of maternity services, inadequate reporting of patient conditions, and a lack of trust between different levels of the system. *“The problem is from both the healthcare providers’ and the clients’ sides. Just the two parties act in a non-harmonized and non-integrated manner.” Key informant interview* participant, program manager.

##### 3.2.4.6 Inadequate transportation infrastructure

Many rural villages are hard-to-reach, primarily due to topographical and seasonal barriers, making maternity services inaccessible. Even nearby rural communities become inaccessible during certain conditions, such as the rainy season when rivers flood and disrupt roads. The challenges in these areas could be addressed through the expansion of transportation infrastructure, including building bridges at large rivers and expanding roads to rural communities. However, the lack of a responsive system to these natural geographical barriers remains a significant deterrent to maternity services. *“I gave my young baby’s birth at home because it was summer time and rivers were so full that we could not pass the rivers to come to the health facility for facility delivery*.” In-depth individual interview participant, end-user.

##### 3.2.4.7 Insufficient monitoring and evaluation

Several theoretically sound PHC programs, including the HEP, PWC, WDA, and home-like MWH, have been implemented in various communities, including hard-to-reach areas. Participants highlight that the functionality of these programs is project-driven, meaning they become fully functional when receiving attention and supportive supervision. However, the current lack of regular supportive supervision, monitoring, and evaluation has led to variations in program functionality. *“Women’s conferences have their own fixed schedules, and the health extension workers are expected to inform and bring all eligible women from the catchment’s area to the conference. But when we midwives go there, they do not act accordingly.”* In-depth individual interview participant, service provider.

In Ethiopia, a number of new recommended delivery approaches, such as the ANC8+ model, HEW-led home-based PNC, and 24-h stay after delivery, have been implemented without piloting or validation tests in the local context. Maternity service providers perceive the ANC8+ model as unreachable and infeasible. They argue that achieving even the ANC4+ coverage targets remains challenging and believe that the new ANC8+ model even adds extra burden. *“However, I think this is too much to maintain all eight ANC contacts’ coverage, as we have not even maintained at least four ANC contacts’ coverage. This model even overburdened us, and policymakers could consider healthcare providers’ perspectives.”* In-depth individual interview participant, service provider.

In Ethiopia, a new home-based PNC program, led by HEWs, has been introduced with a focus on the socio-cultural sensitivity of the postpartum period. However, concerns about the feasibility of the HEW-led home-based PNC arise due to geographical barriers preventing a home-to-home approach. Participants suggest that utilizing WDA leaders for community-based PNC might be more practical, as they are community members with a better understanding of local conditions. *“We programmed that health extension workers are assigned to provide PNC and essential newborn care through a house-to-house approach. But practically, it is hard to do so. Using the women’s development army leaders for community-based postnatal services would be better than anticipating HEWs.”* Key informant interview participant, program manager.

While the policy of a 24-h stay at health facilities after delivery is aimed at optimizing maternal and neonatal health outcomes, implementation gaps are observed. Many postpartum women and their families are not willing to stay at health centers, highlighting the need for further evaluation to develop contextually and culturally compatible implementation strategies. *“Mothers who give birth at health facilities are recommended to stay at least for 24 h after childbirth. Even those mothers who gave birth at health facilities are not willing to stay for 24 h after birth. They come to health facilities just for one purpose, just for giving birth… Some mothers who delivered at health facilities, for example, requested us to allow them to go to their home immediately after giving birth.”* In-depth individual interview participant, service provider.

##### 3.2.4.8 Unsafe security issue conditions

Participants highlighted that widespread unsafe security is a significant factor in the low uptake of maternity services. They pointed to various sources of insecurity, including traditional practices like “blooding,” gangster influence, and internal conflicts. “Blooding,” a traditional retaliatory practice where a family seeks revenge for the killing of a relative, is a deeply rooted source of insecurity. In these cases, if a family member is murdered, the affected family may retaliate by killing the perpetrator or a member of their family. This dangerous security situation, driven by such traditional practices, notably hampers the use of maternity services in some communities. *“If one of the pregnant mother’s family members kills one of the other family members, she obviously cannot attend maternity services as the dead family members hunt her for a revenging purpose.”* In-depth individual interview participant, service provider.

In some communities, gangsters, including armed robbers, pose a significant threat to maternity services. As a result, mothers in these areas are often forced to stop attending maternity services due to the fear of encountering gangsters, especially during the summer season. “*In the summer season, they also frustrate that gangsters will attack them using the harvest as a tentative habitant. This problem is common, especially in June, July, August, and September.”* In-depth individual interview participant, service provider.

In many areas of the study community, unsafe security issues related to internal conflicts have negatively impacted maternity service provision. The effects of these internal conflicts vary: in some cases, ambulance drivers have been threatened or killed due to ethnic conflicts, leading to frustration among other drivers and hindering their ability to serve the community effectively. Additionally, conflicts have damaged significant resources, including ambulance vehicles and other infrastructure, further disrupting the delivery of maternity services. *“One of our ambulance drivers has been killed in connection to ethnic conflicts. Thereafter, other ambulance drivers do not provide ambulance services upon request due to fear of those conflicts and similar death incidences*.” In-depth individual interview participant, service provider.

##### 3.2.4.9 Disrespectful maternity care

Disrespectful care was reported, especially in the chart and laboratory rooms. Compromising mothers’ privacy and confidentiality was a prominent barrier that led women to prefer homebirth and may deter them from seeking continuous maternity care at health facilities. Long waiting times, verbal abuse, and insults persist. Service users of community-based health insurance (CBHI) reported dissatisfaction, perceiving that CBHI members receive only cheaper drugs and laboratory tests at public health centers. They feel ordered to purchase expensive items from private institutions. *“Surprisingly, those patients who are not members of community-based health insurance have accessed and purchased any drug, including expensive drugs, within the health center, as those patients take the drugs directly by purchasing by money. But that is not true for us who are members of* community-based health insurance, *as we do not purchase drugs from the government facilities directly. We are so sorry and disappointed for that matter. We wish we were not members of* community-based health insurance.” In-depth individual interview participant, end-user.

In some health centers, there are not enough midwives to deliver women-centered maternity care effectively. In these situations, a single midwife may handle labor and delivery, ANC, and family planning services all at once. Midwives have reported that they often need to enlist the help of non-healthcare personnel, such as security staff, during labor and delivery, which can compromise the privacy and confidentiality of mothers. *“We midwives are having difficulties providing woman-centered midwifery care as our number in this health center is only two. You can imagine the quality of care while only two midwives provide ANC, delivery care, PNC, family planning service, and so forth. On top of that, one of us gets off, especially in the afternoon, for preparation for night duty. In that case, only one midwife is required to cover all the maternity services. To be honest, I call even non-healthcare personnel like cleaners or security men to assist me while I attend childbirth. I know this is violated care that can compromise women’s privacy and confidentiality. But I have no option that my primary intention becomes just to save women’s lives and to prevent neonatal death*.” In-depth individual interview participant, service provider.

##### 3.2.4.10 Healthcare workers’ dissatisfaction

Healthcare worker dissatisfaction, stemming from non-transparent recruitment procedures, burnout, low wages, and limited opportunities for professional development, has contributed to reduced utilization of the maternity continuum of care. Participants expressed concerns about corrupt practices related to staff transfers, where requests for transfers are often driven by questionable social or financial motivations. This results in significant inequities in the distribution of maternity care providers between urban and rural health centers. Urban health centers frequently have a sufficient or even excess number of midwives, while rural and semi-urban health centers face severe shortages. *“We asked the woreda health office to refill and assign another midwife in place of the transferred one. But the woreda health office replied that they had no budget to do so. Look! If they were genuine, the budget issue would not be an issue as the budget for the transferred midwife is there.”* In-depth individual interview participant, service provider.

Burnout among maternity healthcare workers, including midwives, has led to dissatisfaction and compromised the quality of maternity care. In some health centers, the imbalance between the number of midwives and the number of maternity service users forces a few midwives to handle services throughout the day. This excessive workload leads to burnout, which negatively affects client-provider interactions and overall maternity care quality. *“Of course, there may be flaws in professional ethics related to high workloads… as the number of service users is large, all these could` compromise the client-provider interaction as midwives get exhausted and burned out.”* In-depth individual interview participant, service provider.

Study participants also highlighted that low payment rates, such as professional risk and duty allowance rates, are disparately low compared with the challenging and risky nature of the profession, causing dissatisfaction and demotivation. In addition to the inadequate risk and duty allowance rates, delays in receiving these allowances further contribute to a decline in midwives’ motivation. These low salary and payment rates create challenges for maternity service providers to cope with high living costs and inflation. As a result, healthcare providers may seek additional income sources, compromising the quality of maternity services and potentially exacerbating the already high rates of maternal and child deaths. *“Midwifery is a challenging profession with challenging responsibilities… while she experiences labor pain; we also share the labor pain with her as well. Midwives’ job is unique; you will spend a full night on a single labor case. As compared to this risk, the payment rate is totally incomparable. It is so discouraging.”* In-depth individual interview participant, service provider Participants highlighted a lack of training and continuous professional development opportunities for maternity service providers, leading to demotivation. They emphasized that quality maternity care is unattainable without ongoing professional development and adequate motivational efforts. *“So, unless there are motivational efforts such as continuous professional development, the quality of maternity care is unimaginable.”* In-depth individual interview participant, service provider.

In summary, challenges of MCC within the PHC of northwest Ethiopia exist interdependently across multiple levels—intrapersonal, interpersonal, community, and health facility/system (Figure 2).

## 4 Discussion

This study explored the challenges of MCC within the PHC in northwest Ethiopia, considering both time (critical phases of pregnancy and childbirth) and place (home-community-health facility ecosystem) dimensions. Despite its clinical significance (3, 34), MCC coverage remains low in sub-Saharan Africa (SSA), particularly in Ethiopia (35). Exploring the challenges of MCC through the integration of time and place dimensions provides valuable insights for addressing the multifaceted barriers in maternity care. These barriers span across various levels—intrapersonal, interpersonal, community, and health facility/system—each influencing and being influenced by the others. Understanding these interconnected dynamics is essential for designing effective strategies to improve MCC across all phases of pregnancy and childbirth in PHC settings.

This study finds that low women’s health-seeking behavior (intrapersonal level challenge), lack of family and peer support (interpersonal level challenge), negative cultural influences on maternity care and a lack of community responsiveness (community level challenge), and inadequate health system readiness and response (health facility/system level challenges) contribute to low MCC. The study also revealed several important challenges of MCC at various levels, including misconceptions regarding maternity care, low health literacy and self-efficacy, low perceived quality of care, and mistrust in providers at the intrapersonal level; low autonomy, women’s high workload and responsibilities, peer and family pressure against seeking maternity care, low male involvement, and intimate partner violence at the interpersonal level; cultural influences on pregnancy disclosure, cultural rules of first-childbirth experience, cultural influence on postnatal care, and low social accountability at the community level; and delays in accessing ambulance services, long waiting times for maternity care, shortages of essential healthcare supplies, inconvenient health facility environments, poor coordination of care, inadequate monitoring and evaluation, inadequate transportation infrastructure, unsafe security conditions, disrespectful maternity care, and dissatisfaction among healthcare workers at the health facility/system level.

These challenges highlight the unmet efforts to improve MCC within PHC. Consistent with findings from previous studies (8, 12), these challenges suggest the need for comprehensive interventions mitigating challenges at multiple levels to enhance MCC. Delving deeper, most of the challenges—such as low health-seeking behavior, lack of family and peer support, negative cultural influences, and an unresponsive community—demand integrated efforts focused on raising awareness and creating demand for maternity healthcare. These efforts are likely to be effective through group-based approaches, such as the PWC (36) and the WDA (29), involving key stakeholders, such as end-users, family members and peers, community opinion leaders and representatives, service providers, and program managers. Hence, a sustainable and holistic approach could transform harmful cultural practices into a community that is responsible and accountable for maternity healthcare services, in accordance with standard recommendations. Furthermore, the challenges necessitate targeted interventions that promote family and/or peer support and women’s empowerment and advocate participatory group education, to optimize MCC. In this regard, the World Health Organization (WHO) has recommended context-specific group ANC that incorporates participatory group education and peer support in addition to the usual ANC (37). Aligning with the WHO’s recommendations, we suggest future implementation research within the PHC on group-based health system interventions that foster peer support and group education. This approach could enhance the effectiveness of ANC, an entry gate for MCC, and address the identified challenges, ultimately improving MCC and maternal and neonatal health outcomes (37).

The findings in this study highlights that there are still ongoing incidents of intimate partner violence (IPV) in the community, and the victims have no opportunity to attend for maternity services. This finding is in accordance with previous studies conducted elsewhere (38–40) and suggests that facility-based violence screening is not sufficient to trace the problems. Hence, we recommend integrated health facility- and community-based violence surveillance and responses based on the WHO recommendation (37), and further research for the implementation strategies.

The features of the lack of health system readiness and response—such as delays in accessing ambulance services, excessive waiting times for maternity care, shortages of essential healthcare supplies, lack of coordination for maternity care, inadequate transportation infrastructure, unsafe security conditions, disrespectful maternity care, and healthcare workers’ dissatisfaction—elucidate the unmet PHC performances against the 2025 Ethiopian national targets of the second health sector transformation plan (HSTP-II) (2). These targets include service coverage improvement (i.e., ANC4+ from 43 to 81%, facility delivery from 48 to 76%, and early PNC from 34 to 76%) and mortality reduction (i.e., MMR from 401 to 279 per 100,000 live births and NMR from 33 to 21 per 1,000 live births) by 2025. The findings are also too concerning to reach the global 2030 SDG 3 targets (1). These findings contradict the core principles of PHC, including accessibility, people-centeredness, coordination, comprehensiveness, continuity of care, and quality of care (16). These findings are also incompatible with the 14 strategic directions of the Ethiopian HSTP-II (2). Supported by previous studies (41, 42), these findings suggest the need for coordinated efforts and good governance and leadership capable of ensuring the availability and accessibility of quality maternity care in a sustainable and timely manner. Hence, stakeholders involved in PHC programs need to ensure the sustainable accessibility of healthcare inputs (through timely refilling of stockouts), shorten the time to access ambulance services through a decentralized system, tackle the misuse of ambulance cars, and improve ambulance drivers’ incentives and their numbers.

The study reveals low healthcare monitoring and evaluations in Ethiopia, leading to the direct adoption of new maternity delivery approaches without contextual validation, contradicting the Ethiopian PHC system’s assumption of community needs and cultures (2). This study highlights significant concerns about the acceptability and feasibility of implementing the new ANC8+ model in Ethiopia. Service providers have expressed doubts about the feasibility of this model, which the Ethiopian Ministry of Health has endorsed and implemented nationwide since 2022. They are frustrated, noting that even the less ambitious ANC4+ targets have not been met, and fear that the new model will impose additional burdens on both mothers and providers. These concerns are critical, as the success of the new model depends on its acceptance by both service users and providers. Without timely evaluation and corrective measures, the model may face challenges similar to those observed in other LMICs, where the uptake of ANC8+ is generally low (43). For instance, data from LMICs show that the pooled prevalence of ANC8+ is just 13%, with a range from 1% in countries like Senegal, Uganda, and Zambia to 74% in Jordan. This indicates that full coverage of eight ANC contacts is quite limited in many countries in SSA, including Senegal, Uganda, Zambia, Mozambique, Mali, Guinea, Cameroon, and Benin (43). These findings, combined with the current study’s results, underscore the urgent need for a thorough implementation evaluation of the ANC8+ model starting from the very beginning in Ethiopia.

This study’s finding highlighted the sub-optimal woman-centered care, evidenced by disrespectful maternity care. Disrespectful care in our study was manifested through a lack of privacy and confidentiality, denial of preferences, verbal and physical abuse, and perceived discriminatory practices. Such mistreatment can severely compromise the quality of care, which is essential for achieving universal health coverage and includes respectful maternity care (RMC) as a core component. When women feel supported, respected, safe, and involved in shared decision-making with their providers, they are more likely to uptake MCC and have positive childbirth experiences (44). However, the study highlights that disrespectful care creates a psychological barrier between women and maternity service providers, discouraging women from utilizing MCC within the PHC system in northwest Ethiopia (41). Beyond the implications for quality of care disrespectful care is an important human rights issue (45) as all women have the right to be free from harm, ill-treatment, and to have their choices and preferences respected (46). The study further revealed that disrespectful care often originated from the chart and laboratory rooms, highlighting the need to address the behavior of staff members in these areas to ensure respectful care and optimize MCC. In addition, since perinatal women’s values related to respectful care can vary, it is crucial for maternity healthcare providers to inquire about women’s values, needs, and fears, and provide appropriate support. Therefore, improving woman-centered care through strengthening the midwifery model of care and respectful maternity care training should be a top priority.

Dissatisfaction among healthcare providers, evidenced by burnout, low duty and risk allowance rates, non-transparent healthcare worker deployment procedures, and low opportunities for capacity building and professional development, has emerged as a significant challenge at the health facility and system levels for MCC within PHC in northwest Ethiopia. This suggests problems with human resource management (HRM) and handling. These issues are particularly concerning given the HSTP-II’s goal of ensuring an equitable distribution of a skilled, motivated, and compassionate workforce to deliver quality health services (2). The findings cast doubt on the progress of the Ethiopian PHC system toward the 2025 HSTP-II targets (2). Conducted midway through the HSTP-II timeline, this study signals the need for a nationwide review of current PHC practices against HSTP-II objectives and the implementation of timely corrective measures. Hence, stakeholders need to work collaboratively to overcome those sources of dissatisfaction among maternity healthcare workers which compromise the quality of maternity care. We further recommend stakeholders to: (1) overcome unsatisfactory HRM through decreasing non-transparent maternity service providers’ recruitment procedures and burnout; (2) optimize healthcare providers’ retention mechanisms through improving incentives and risk allowances; and (3) advance healthcare providers’ capacity and competency through strengthening platforms for continuous professional development.

### 4.1 Limitations

This study may have certain limitations that readers need to consider. The article primarily reports on the challenges, despite the presence of numerous opportunities for enhancing the MCC within the PHC system in northwest Ethiopia. Due to the richness of the data, a separate article has been prepared to discuss the opportunities for MCC enhancement. Additionally, the findings largely rely on participants’ recollections of their past experiences, which may limit the relevance of the results to current circumstances. To mitigate this bias, the researchers conducted iterative in-depth interviews with probing techniques. We encountered challenges when mapping themes and sub-themes onto the SEM, particularly in distinguishing between health facility-level issues and broader health system/policy-level challenges. This underscores some limitations of the SEM, although it is adaptable to different study needs (9, 11). We further adapted the model by placing the community level ahead of health facility levels to better capture MCC challenges in the home-community-health facility continuum. While this adjustment suits the study context, the SEM’s flexibility allows it to be tailored for various research settings (9, 11). Another limitation is the potential influence of the researchers’ perspectives during data collection and interpretation. Additionally, contextual factors may have impacted the applicability of the findings. The data were collected around the time of internal conflict, which may have disrupted healthcare services and altered participants’ maternity care experiences.

We took several steps to address the study’s limitations and ensure the trustworthiness of the findings. First, the primary author spent several months in the field, demonstrating prolonged engagement to build a comprehensive understanding of the local context (47–49). To foster openness, data collectors reassured participants about the absence of right or wrong answers, emphasized their autonomy, and assured confidentiality and used iterative questioning to enhance data depth, resulting in rich and detailed insights (47). We also used triangulation to enhance credibility, dependability, and confirmability (47). This involved gathering data from various sources, including end-users, significant others, community representatives, service providers, and program managers. Researcher reflexivity was clearly outlined to ensure confirmability, reflecting the researchers’ roles and potential influences throughout the study. To improve transferability and dependability, we provided a thick description (31) of the study’s context, methodology, and analytical processes, offering detailed documentation of the research setting. Finally, the use of direct participant quotes to illustrate key themes added to the authenticity of the findings by grounding the results in participants’ real experiences. These collective efforts helped ensure that the study’s findings were rigorous, reliable, and reflective of the participants’ realities.

## 5 Conclusion

The challenges of MCC within the PHC in northwest Ethiopia are multifaceted, including low women’s healthcare-seeking behavior (intrapersonal level challenge), lack of family/peer support (interpersonal level challenge), negative cultural influences on maternity care and a lack of community responsiveness (community level challenge), as well as inadequate health system readiness and response (health facility/system level challenges), all highlight the unmet efforts to improve MCC within PHC. Hence, interventions aiming to improve the MCC within PHC in northwest Ethiopia require a multi-faceted approach that addresses challenges across intrapersonal, interpersonal, community, and health facility/system. Introducing, contextualizing, and integrating evidence-based multifaced approaches (e.g., group ANC approach) with the existing PHC system may: (1) enhance health literacy and promote health-seeking behavior, (2) strengthen family and peer support, and (3) address cultural barriers and foster community engagement in maternity services, thereby mitigating those multilevel challenges of MCC. Moreover, improving health system readiness and response for maternity services is warranted. Therefore, maternity service stakeholders should work collaboratively to: (1) increase investments in healthcare infrastructure, ensuring that health facilities are well-equipped with essential maternity supplies, motivated and skilled staff, and emergency transportation services; (2) conduct regular maternity program monitoring and evaluation and take timely corrective interventions; and (3) address security issues.

## Data Availability

The raw data supporting the conclusions of this article will be made available by the authors, without undue reservation.
