# Supplementary material for: Challenges of maternity continuum of care within the primary health care in northwest Ethiopia: interpretive description using a socio-ecological model
Source: Front Public Health. 2024 Dec 11;12:1401988. doi: 10.3389/fpubh.2024.1401988 (PMC11668779; doi:10.3389/fpubh.2024.1401988)
Supplement: Supplementary file 2 [file Table_2.DOCX]

**S2 File. Focus group discussion interview guideline for challenges of maternity continuum of care**

S1 Table.

**1.1 Information sheet**

College of Medicine and Health Sciences & Specialized hospital, University of Gondar, Ethiopia

**Research title:** Challenges of maternity continuum of care within the primary health care of northwest Ethiopia

**Principal investigator**: Mr. Muhabaw Shumye Mihret (department of clinical midwifery, University of Gondar); Tel: 251 918473798; Email: [muhabawshumye@ gmail.com](mailto:muhabawshumye@gmail.com)

**Co-investigators**: Professor Kassahun Alemu, Dr. Debrework Tesgera, Dr. Lemma Derseh Gezie, Professor Kerstin Erlandsson and Professor Helena Lindgren

**Introduction**: Good morning/ Good afternoon. My name _________ (the main data collector and research assistants should introduce their name one by one). On behave of the research team mentioned above; we are here to collect data for the research as entitled above. The study granted ethical clearance from university of Gondar and permission letter from Amhara regional health bureau, central Gondar zonal department and your Woreda health office to conduct this study. I am going to give you information related to the study and invite you to be part of this research. You need to confirm your participation whether or not you will participate in the research. Now we are going to read basic information about the study and your participation/non-participation. You can ask us any question after information is provided or at any time. Shall we proceed to the basic information? (Get permission to provide the basic information about the study and their participation/non-participation).

**Purpose of the research**: to explore potential opportunities for maternity continuum of care improvement as entitled above and the findings will be used to inform policy and practices for the betterment of maternity continuum of care and maternal, neonatal and child health in the study area and in Ethiopia as well.

**Voluntary participation**: participation in this research is entirely voluntarily; even you can change your mind at any time after deciding early.

**Benefit of the study**: study partcipant will have benefits from the result of the study which will use to inform policy and implement maternity health service programmes and will be beneficiary for the study partcipants as well as the overall community.

**Risk of study**: participating in this study will have no any risk or harm except your time.

**Right of study participants**: you have full right to partcipate or not to partcipate as well as you may end the interview after enrolled to partcipate at any time. You can ask any question you want at any time. You are also kindly requested to respect other’s idea and share with your idea freely and genuinely.

**Confidentiality**: any information obtained from you will be maintained confidential and we will not write personal identification such as name. The data will be used only for this study. We researchers kindly would like to assure that we are independent and autonomous to handle any information you told us firmly confidential and use for the study purpose only. Moreover, the ideas to be discussed here are not sensitive, rather they are commonly shared experiences, feelings, practices, perceptions etc. In addition, each of you will have a code and we should use the code during the discussion, not the name of the discussant inorder to maintan particiapnts’ anonymity throughout the focus group discussions (FGD) as we will use audio taping based on your informed consent.

**Duration of interview**: It will vary depending on the issue rasied and our discussions. It might take 40-70 mnutes in average.

**Incentive /payment**: you will not have got any payment for partcipating in this study.

**Person you contact** : this research project reviewed by Institutional Review Board (IRB) of University of Gondar. If you will have any concern you can contact one of the concerned bodies and you may ask at any time you want.

**Audio taping**: the study good to be audio taped with full informed consent. The recorded audio recordings will be handled firmly confidental and will not be shared with any other third person or party. Particpation without audio taping is also possible.

**Principal investigator:**

Name: Mr. Muhabaw Shumye Mihret (School of Midwifery, University of Gondar); Tel: +251 918473798; Email: [muhabawshumye@gmail.com](mailto:muhabawshumye@gmail.com)

Are you willing to participate in the interview and stay with us for a few minutes (40-70) now? [ ] Yes, go to the informed consent sheet section [ ] No, thank the individual and proceed to next eligible participant

**1.2 Informed** consent form

I am informed that my identity will be handled anonymously and the information I offer will be treated confidentially. I have also been informed that I can refuse to participate in the study or not respond to questions if I am not interested. Furthermore, I have been informed that I can stop responding to the questions at any time in the process. I am informed that my participation, non-participation, or refusal to answer questions will not have any effect on my life. I am informed that no financial incentives will be given for my participation in the study. I am also informed that my response will be used to develop strategies that help for the programs on maternity continuum of care. I have read this form or it has been read to me in the language I realized and understand all information stated above.

Are you willing to participate in this study?

If respondent agrees to be interviewed, remind them to put the signature and assure their permission for audio taping.

Focus group discussion’s code No.:______ Date__________

| S.no. | Participant’s code | Signature | Phone number if any |
| --- | --- | --- | --- |
| 1 |  |  |  |
| 2 |  |  |  |
| 3 |  |  |  |
| 4 |  |  |  |
| 5 |  |  |  |
| 6 |  |  |  |
| 7 |  |  |  |
| 8 |  |  |  |
| 9 |  |  |  |
| 10 |  |  |  |
| 11 |  |  |  |
| 12 |  |  |  |

Starting time_______________ End time___________________________

Main interviewer‘s name---------------------------Signature-------------------Date---------------------

Note taker’s name: ---------------------------Signature-------------------Date-------------------

Facilitators’ name: ---------------------------Signature-------------------Date-------------------

**1.3 Sociodemograhic variables**

| Participants’ code | Age | Gender | Occupation | Marital status | Major Representative category* | Status in the reproductive cycle** | Special role within the primary healthcare*** |
| --- | --- | --- | --- | --- | --- | --- | --- |
|  |  |  |  |  |  |  |  |
|  |  |  |  |  |  |  |  |
|  |  |  |  |  |  |  |  |
|  |  |  |  |  |  |  |  |
|  |  |  |  |  |  |  |  |
|  |  |  |  |  |  |  |  |
|  |  |  |  |  |  |  |  |
|  |  |  |  |  |  |  |  |
|  |  |  |  |  |  |  |  |
|  |  |  |  |  |  |  |  |
|  |  |  |  |  |  |  |  |
|  |  |  |  |  |  |  |  |

*Major Representative Category: a) End-service user, b) Significant others, c) Community representative; **Status in the reproductive cycle: use a) Pregnant, b) Postnatal, c) Non-perinatal, d) not applicable (male); ***Special role within the primary health care: a) Leader of women’s development army, b) Traditional birth attendant, c) Midwifery practitioner, d) Health extension worker, e)Other community’s opinion leader, f) Service-user or significant other (with no additional special role)

**1.4 Questions/probes to explore** Challenges of maternity continuum of care

1. What do you think why women discontinue maternity services utilizations after booking for ANC? Probe: opinion on

a) Quality of care,

b) Waiting time,

c) Healthcare system (infrastructure, healthcare provider, and service availability healthcare providers’ commitment)

D) Barriers within home-community-health facility ecosystem

1. What challenges of have women experienced at each phase in the home-community-health facility/system ecosystem to receive maternity services in a continuum manner? (consider the following probing points if applicable)

a) At home (women’s readiness and their social network (husbands, elder woman or men (including mother-in-laws, peers…)

b) At the community (Cultural and traditional beliefs and practices, community representatives’ responsiveness to maternity services, community volunteers, government bodies, civic societies, etc)

c) At the health facility/system (Perspectives from maternity services providers, lab service providers, pharmacy healthcare workers, charting service providers, medical inputs such as inputs for lab service, pharmacy service, etc.

d) Transportation-related issues (Ambulance readiness)

1. What efforts had you/others made whenever you/others face challenges in providing/receiving maternity continuum of care?
2. What do you wish to be corrected to overcome those challenges of maternity services you mentioned?
3. Do you have any idea you want to add?

Thank you so much
